# First evidence of a palaeo-nursery area of the great white shark

**DOI:** 10.1038/s41598-020-65101-1

**Published:** 2020-05-22

**Authors:** Jaime A. Villafaña, Sebastian Hernandez, Alonso Alvarado, Kenshu Shimada, Catalina Pimiento, Marcelo M. Rivadeneira, Jürgen Kriwet

**Affiliations:** 1 University of Vienna, Department of Palaeontology, Althanstraße 14, Geocenter, 1090 Vienna, Austria; 2 Centro de Investigación en Recursos Naturales y Sustentabilidad, Universidad Bernardo O’Higgins, Santiago, Chile; 3 Biomolecular Laboratory, Center for International Programs, Universidad VERITAS, 10105 San José, Costa Rica; 4 Sala de Colecciones Biológica, Facultad de Ciencias del Mar, Universidad Católica del Norte, Coquimbo, Chile; 5 Department of Environmental Science and Studies and Department of Biological Sciences, DePaul University, Chicago, Illinois 60614 USA; 6 Sternberg Museum of Natural History, Hays, Kansas 67601 USA; 7 Department of Biosciences, Swansea University, Swansea, SA28PP United Kingdom; 8 Smithsonian Tropical Research Institute, Balboa, Panama; 9 Laboratorio de Paleobiología, Centro de Estudios Avanzados en Zonas Áridas (CEAZA), Coquimbo, Chile; 10 Departamento de Biología Marina, Facultad de Ciencias Biológicas, Universidad Católica del Norte, Larrondo, 1281 Coquimbo, Chile; 11 Departamento de Biología, Universidad de La Serena, Av. Raul Bitrán, 1305 La Serena, Chile

**Keywords:** Palaeontology, Marine biology

## Abstract

Shark nurseries are essential habitats for shark survival. Notwithstanding the rich fossil record of the modern great white shark (*Carcharodon carcharias*, GWS), its use of nursery areas in the fossil record has never been assessed before. Here, we analysed the fossil record of the GWS from three South American Pliocene localities, assessed body size distributions and applied previously established criteria to identify palaeo-nurseries. We found that juveniles dominate the Coquimbo locality (Chile), whereas subadults and adults characterize Pisco (Peru) and Caldera (Chile), respectively. These results, summed to the paleontological and paleoenvironmental record of the region, suggest that Coquimbo represents the first nursery area for the GWS in the fossil record. Our findings demonstrate that one of the top predators in today’s oceans has used nursery areas for millions of years, highlighting their importance as essential habitats for shark survival in deep time.

## Introduction

Shark nursery areas are essential habitats where young are born or reside, and where their growth is facilitated^1^. Generally, nursery areas are defined by the following criteria: there is higher relative abundance of juveniles and neonates compared to others areas; immature sharks must show a tendency to return and stay for long periods of time; they are used by immature sharks over years^1,2^; and they are geographically discrete zones that provide two main benefits: protection from predation and abundant food resources^1^. Although these criteria have been useful to identify modern nurseries^3–11^, some of them can be rather difficult to apply to the fossil record. Accordingly, nurseries areas in the geological past (herein palaeo-nurseries) have been proposed based on the criteria for modern species, but adapted to the fossil record (1) water-depth: they are shallow-water habitats hence offering young with protection from larger predators^12^ (2) productivity: they are highly productive habitats providing abundant resources and facilitating growth^2^ (3) preponderance of young individuals: they are heavily dominated by juveniles and neonates^13–17^. Identified shark palaeo-nurseries areas span widely chronologically, geographically, and taxonomically, e.g., hybodontiforms and xenacanthiforms from the late Triassic of Kyrgyzstan; lamniforms from the Palaeocene of South Carolina, USA; *Otodus* from the late Miocene of South Carolina; *O. megalodon* from the late Miocene of Panama; and *Carcharhinus brachyurus* from the late Miocene of Peru^13–17^. The finding of nursery areas in the fossil record suggests they have been essential habitats for some shark species throughout evolutionary time.

Nursery areas are considered to be crucial for the recovery and persistence of shark populations^18^. More recently it has been demonstrated that nursery areas are of utmost importance for maintaining sustainable breeding populations^19–21^. These have large and long-lasting effects on population size, secure the survival of shark species and influence the distribution of populations^21^. Additionally, these areas also limit the access of larger sharks and therefore decreasing the predation risk^2^. As such, the identification of nursery areas has significantly increased in recent years in an effort to mitigate of declines in shark numbers resulting from various anthropogenic activities and their impacts^1^.

The great white shark (herein, GWS) is a large, cosmopolitan, top predator^22^ and comprises six genetically distinct population from Australia/New Zealand, South Africa, Mediterranean, North West Atlantic, North East Pacific, and Japan^23^. It is also a highly migratory species that inhabits a wide range of marine environments, from very shallow waters of the continental shelf to oceanic waters and around remote islands, tolerating temperatures from 5° to 25 °C^24^. Its vertical distribution typically ranges from 0 to 250 m, but can extend down to 1200 m in some cases^25^. This species has been documented in aggregations around rocky reefs near pinniped colonies in northern California, eastern Australia, Canada and South Africa^26–28^. Its presence along south-eastern Pacific coasts is uncommon^29–33^. The GWS is a generalist feeder with increasing trophic levels through ontogeny^34^. Young-of-the-year (YOY; <175 cm) and juveniles (JWS; >175–300 cm) generally feed on teleost fishes, invertebrates and others sharks, whereas subadults and adults (>300 cm) commonly feed on aquatic mammals^35^. Despite its importance as apex predator for the stability of marine ecosystems, the GWS is currently considered vulnerable to extinction due to increased pressure in fisheries^36^. Current knowledge on GWS nursery areas is limited. However, several nurseries have been proposed from different regions around the world. For instance, in North America two large nursery area extends from the Southern California Bight (Point Conception to San Diego) to the Baja California Peninsula and from the New York-New Jersey Bight^3–6^. In addition, Bahia Sebastian Vizcaino (off central Baja California) has recently been identified as another important GWS nursery area^7^. In Europe, the areas around the Sicilian Channel and the Aegean Sea are reported as zones with high abundances of juveniles^8,9^. In the southern hemisphere, three nurseries areas have been reported from Australian and South African waters^10,11^.

The GWS has a rich fossil record^37,38^, but palaeo-nurseries have not yet been reported for this species. Here, we present the first evidence of palaeo-nursery areas and the body size distribution for the GWS from the Pliocene fossil record, specifically from Peru (Pisco Formation^39^) and Chile (the Bahia Inglesa and Coquimbo Formations^40–42^). Assessing the presence of its palaeo-nurseries could unravel more details about the evolutionary history the GWS in the south-eastern Pacific and in general, improve our understanding of past diversity and distribution patterns of apex predators.

## Results

In total, we identified 48% (113 of 234) of teeth as laterals, followed by anterior (41%, 97 of 234) and intermediates (9%, 22 of 234). The most posterior teeth (L5 to L7) were less abundant amounting to only 15% (17 of 113) in all sites. In terms of the body size, our results showed TL ranging from 155–729 cm (Supplementary Fig. 2; Supplementary Dataset 1). Records from Caldera, Chile (27°S), included the largest specimen whereas the smallest individuals come from Coquimbo (29°S).

Our results show that the body size distribution for the GWS was significantly different among localities (Kruskal-Wallis test, p < 0.0001; a posteriori test, p < 0.006 between each pair of localities), with a smaller median value for Coquimbo (271 cm), followed by Pisco (350 cm) and Caldera (411 cm). While GWS juveniles dominated the Coquimbo population (Table 1), subadults were most common in Pisco and Caldera. Additionally, the others size categories also vary across localities (Table 1; Fig. 3). YOY are very rare in Coquimbo and Caldera, and are absent from Pisco. JWS occurred in the highest numbers in Coquimbo, followed by Pisco and Caldera. Based on the length of maturity (≥480 cm for females, ≥360 cm for males and ≥450 for undetermined sex)^43,44^, the highest number of adults occurs in Caldera, followed by Pisco and Coquimbo. Finally, Gaussian Mixture Modelling indicates the presence of two body size clusters each in Pisco and Coquimbo. In Pisco, the clusters indicate specimens above the size for JWS and the length of subadults and adults (Fig. 3a; Supplementary Fig. 3). In Coquimbo, the clusters indicate specimens within the body size range for JWS and the length of subadults females and adult males (Fig. 3c; Supplementary Fig. 3). In Caldera, only a single cluster is detected, the cluster indicates specimens within the length of subadults females and adult males (Fig. 3b).

**Table 1:** Body size distribution of Young-of-the-year (YOY), juveniles (JWS), subadults and adult individuals of the Great White Shark (GWS) from the lower Pliocene of Pisco (n = 80), Caldera (n = 85) and Coquimbo (n = 69).

Region	N° of specimens	% of specimens
**(A) YOY (120 to 175 cm)**		
Pisco	0	0
Caldera	1	1
Coquimbo	2	3
**(B) JWS (175 to 300 cm)**		
Pisco	21	26
Caldera	11	13
Coquimbo	42	61
**(C) subadult males (≥300 to 360 cm)**		
Pisco	50	63
Caldera	49	58
Coquimbo	20	29
**(D) subadult females (≥300 to 450 cm)**		
Pisco	28	35
Caldera	21	25
Coquimbo	10	14
**(E) adult males (≥360)**		
Pisco	31	39
Caldera	53	62
Coquimbo	15	22
**(F) adult females (≥480)**		
Pisco	9	11
Caldera	22	26
Coquimbo	4	6
**(G) adults undetermined sex (>450)**		
Pisco	9	11
Caldera	25	29
Coquimbo	5	7

**Figure 1:**
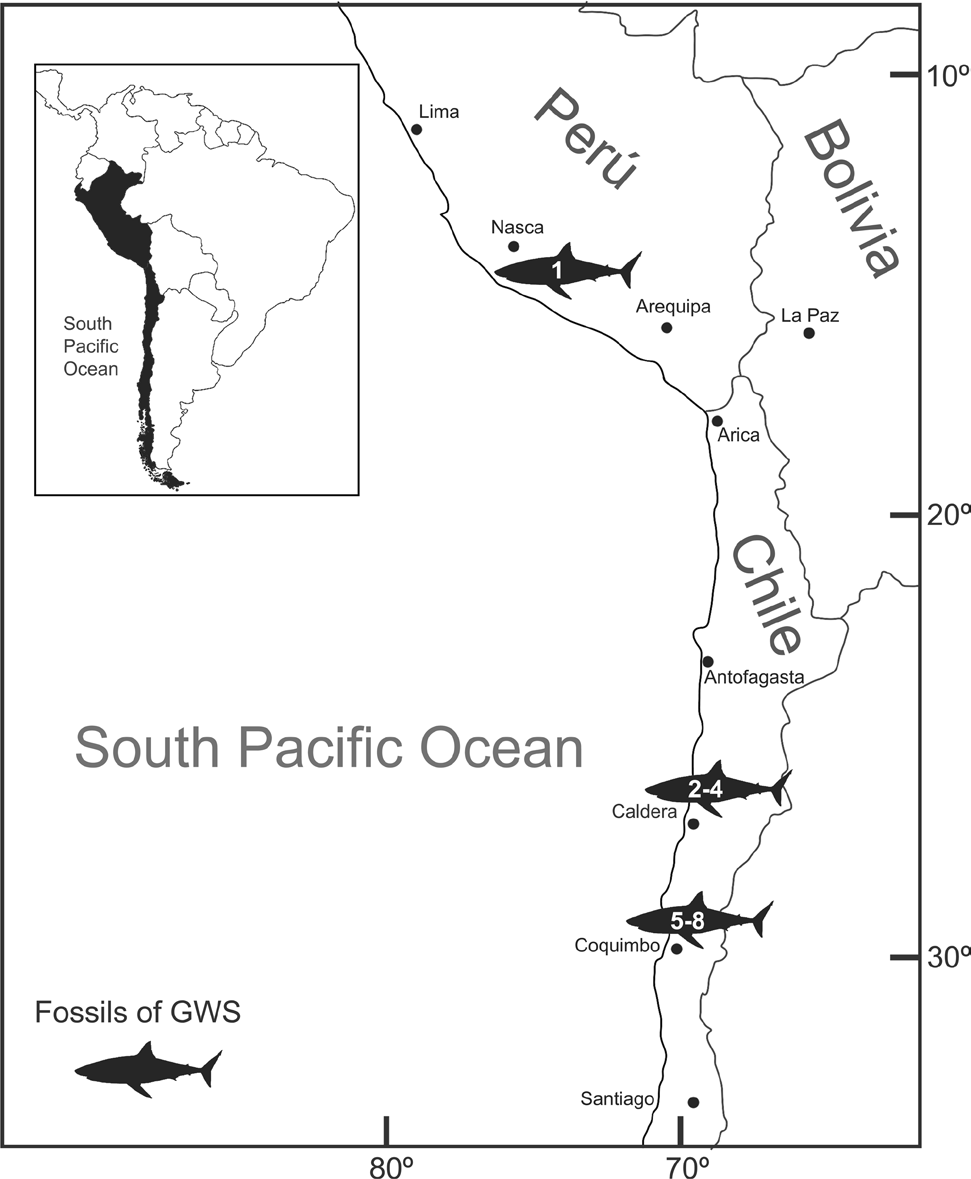
Map showing localities of the fossil Great White Shark (GWS) from the lower Pliocene of the eastern Pacific of South America. 1, Sacaco; 2, Caldera; 3, Mina Fosforita; 4, Norte Bahia Caldera; 5, Quebrada Camarones; 6, La Cantera Baja; 7, Quebrada Las Rosas; 8, La Herradura.

**Figure 2:**
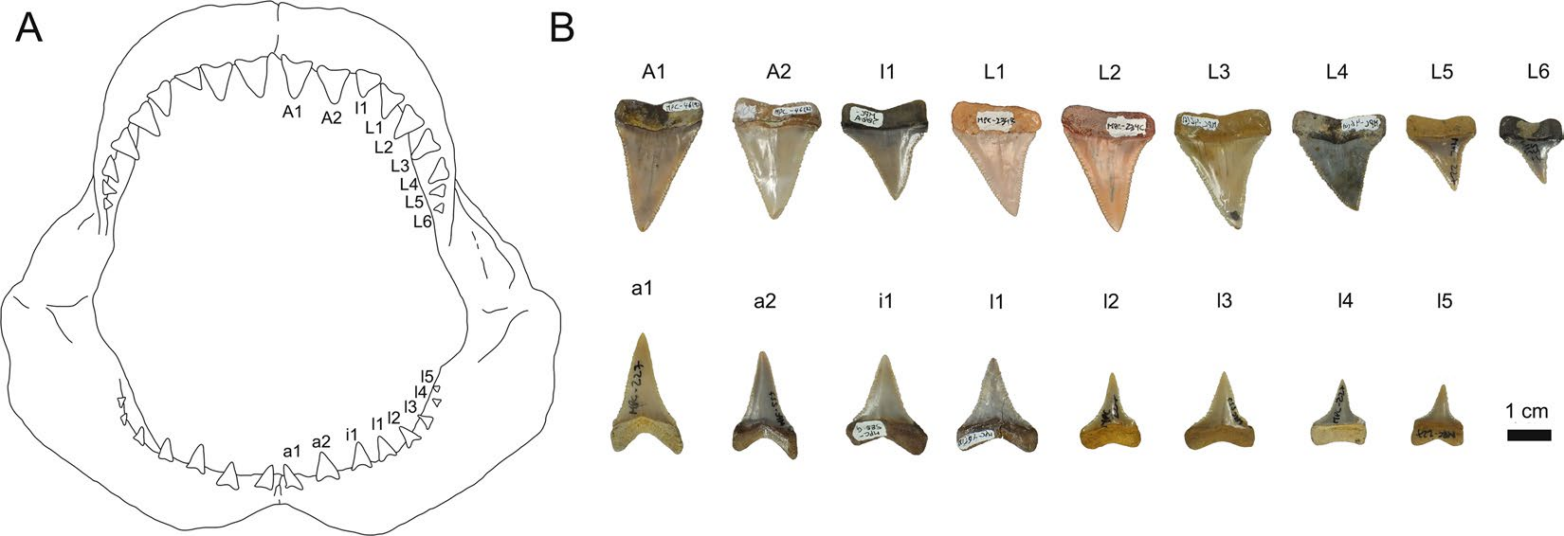
(**A**) Representation of tooth position within the jaw of the extant Great White Shark (GWS), (**B**) selected fossil specimens for each tooth position.

**Figure 3:**
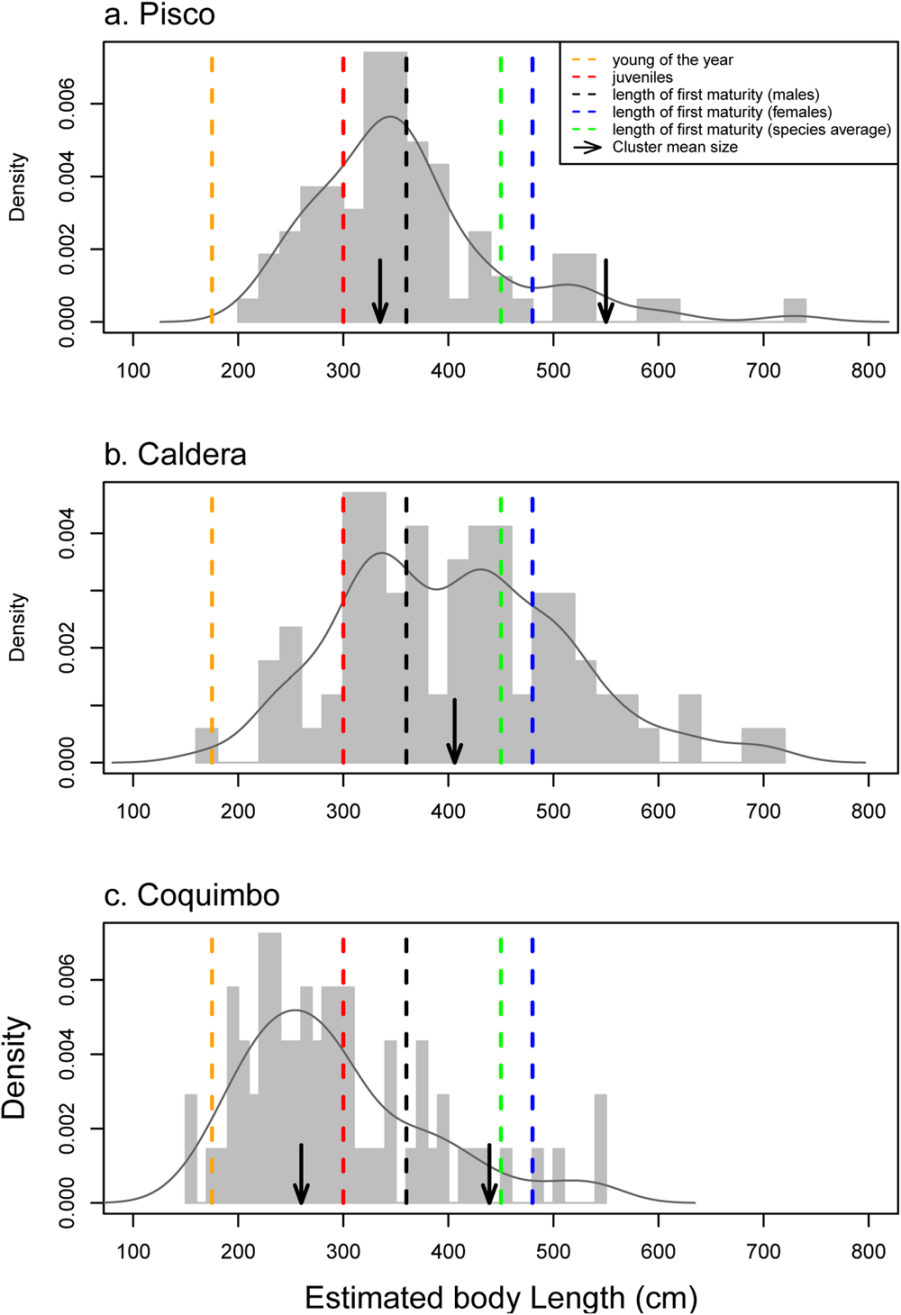
Frequency distribution of the estimated body length of GWS from (**A**) Pisco, (**B**) Caldera and (**C**) Coquimbo. Dashed lines represent the length of young of the year, juveniles, first maturity for males, females and species average.

## Discussion

**Figure 4:**
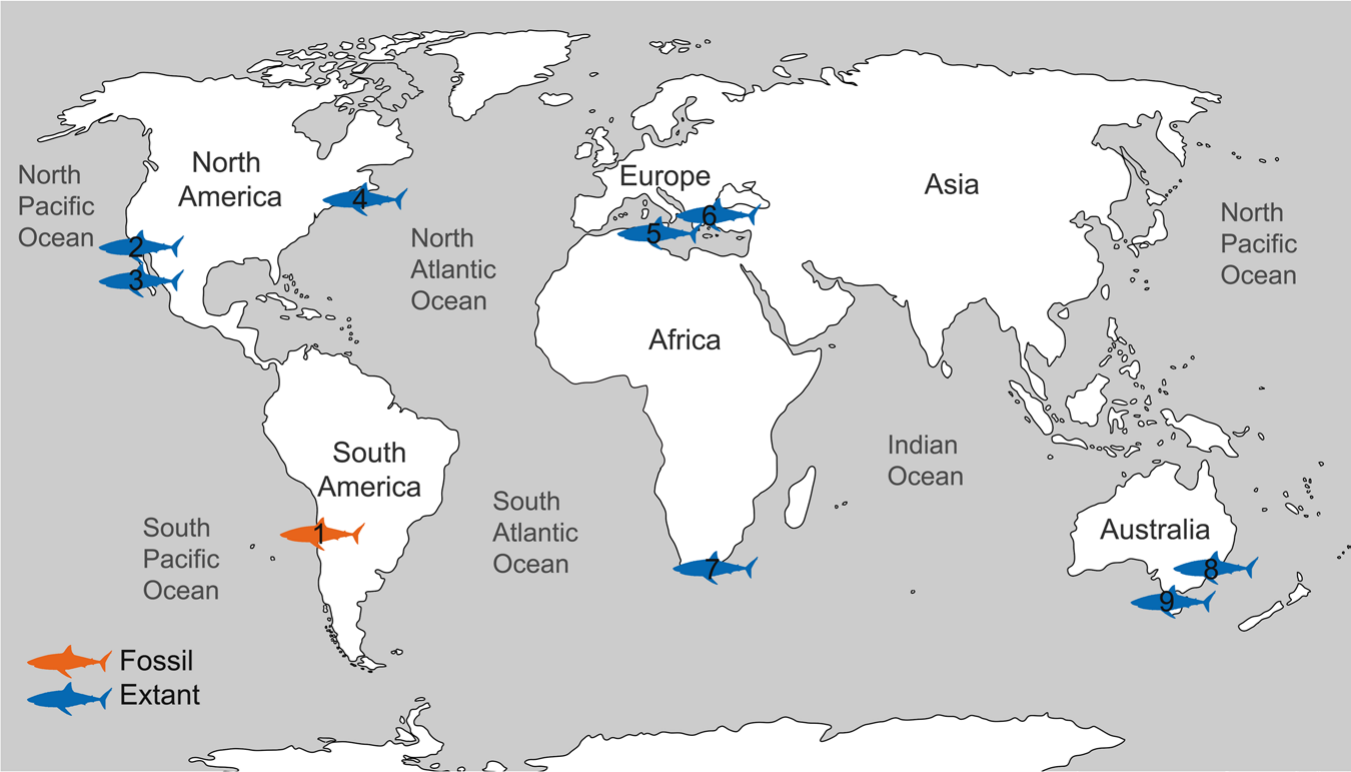
Extant (blue) and fossil (orange) nursery areas of the Great White Shark (GWS). 1, Coquimbo (this study); 2, Southern California Bight^3–5^; 3, Bahia Sebastian Vizcaino^7^; 4, New York-New Jersey Bight^6^; 5, Sicilian Channel^8^; 6, Aegean Sea^9^; 7, Algoa Bay^10^; 8, Port Stephen^11^; 9, Cornet Inlet^11^.

The GWS is the second most abundant chondrichthyan in the Pliocene of the eastern Pacific of South America (22%, 68 of 313 all fossil species occurrences in the region)^45^. However, this study represents the first attempt to assess the body size distribution and to identify possible palaeo-nursery areas for this species (Fig. 4; Supplementary Fig. 2). Based on the main criteria to define palaeo-nursery areas (high frequency of juveniles, food availability, and shallow-water depths)^2^ and the fact that YOY and JWS occur in similar locations and thus may share similar habitat requirements^44^ (Table 2), we propose Coquimbo as the first nursery area for the GWS in the fossil record.

**Table 2:** Criteria used to define a palaeo-nursery area for each locality.

Locality	Bathymetry	Food availability	Dominated by juveniles
Pisco	Shallow-Upper shelf^52^	whales, phocids, dolphins, large and small fishes (chondricthyans and bony fishes)^38,52^	No
Caldera	Shallow-Upper shelf^57^	whales, dugongids, dolphins, phocids, large and small fishes (chondricthyans and bony fishes)^41,54–56^	No
Coquimbo	Shallow-Upper shelf^46,47,49–51^	whales, large and small fishes (elasmobranchs and bony fishes)^40,42,46,47^	Yes

The Coquimbo locality not only displays the highest percentage of JWS and the lowest percentage of subadults and adults (for both males and females: Table 1; Fig. 3c), but it is also characterized by a high abundance of other fish taxa (e.g., *Heterodontus*, *Squalus*, *Myliobatis* and *Euthynnus lineatus*)^40,42,46,47^ which represent potential prey for JWS^35,48^. In terms of water depth, the Coquimbo Formation has been interpreted as a shallow marine environment based on the presence of typically shallow-water invertebrates (e.g., *Crassostrea* and *Incatella*) and vertebrates (e.g., *Thalassocnus* and *Heterodontus*)^46,47,49–51^ (Table 2). The nursery area of Coquimbo thus would have offered to JWS abundant food resources as well as protection from larger predators.

In Pisco, our estimations indicate the highest frequency of subadults, absence of YOY and the presence of significant number mature adults (Table 1; Fig. 3a). Studies have shown that *C. carcharias* from Pisco formation cohabited with a high abundance of marine mammals as phocids, dolphins and whales^39,52^, being the target prey for larger subadults and adults GWS^34,35^. In terms of water depths, the depositional conditions of Pisco Formation have been interpreted as shallow to deep platform water, based on the presence of diatom and radiolarian taxa^53^. Given the absence of juveniles, the high abundance of marine mammals, and the shallow and deep-water depths (Table 2), we propose Pisco not as a nursery, but as a feeding area where subadult GWS had already switched their dietary preferences to marine mammals^34^.

Caldera displays the lowest percentage of JWS and the highest percentage of mature GWS (Table 1 and Fig. 3b). This locality is also characterized by high abundance of fossil marine mammals (phocids, dugongids, dolphins and whales)^41,54–56^. Furthermore, this locality is known for having a high abundance of the top predator *O. megalodon*^45^, which could be a potential predator for young GWS. Based on benthic foraminifers, the depositional environment of Bahia Inglesa formation has been interpreted as ranging from the littoral zone to the upper continental slope^57^. Considering the low percentage of JWS, the dominance of adults, the purported deep-water conditions, the high abundance of marine mammals and the presence of potential predators for young GWS (Table 2), Caldera seems to have been preferred by mature GWS and therefore may represent a feeding habitat.

Two other palaeo-nursery areas have been described in detail for other Neogene sharks from America^16,17^ following the three criteria used here. Pimiento *et al*.^16^ proposed the first evidence of a nursery area for *O. megalodon* from the late Miocene Gatun Formation in Panama. Landini *et al*.^17^ suggested a nursery area for the copper shark, *C. brachyurus* in the late Miocene of Peru (Pisco Formation). The nursery area that we proposed here displays a lower, yet comparable abundance of juveniles relative to the previously described nurseries (61% in Coquimbo *vs*. 88% in Panama *vs*. 84% in Peru: Table 1). The characterization of Pisco as a nursery area for *C. brachyurus* is surprising given the occurrence of possible predators.

The Pliocene epoch (5.33‒2.58 Ma), when it was comparably warmer than today^58,59^, has been proposed as the closest analog to anticipate the effects of ongoing greenhouse climatic warming^60,61^. Our results suggest that warmer sea surface temperatures may favor the existence of novel nurseries at currently temperate areas, re-shaping population dynamics and connectivity of the GWS with cascading efforts on local food webs. Thus, some areas may become new targets for marine conservation effort. New studies aimed at describing the location of past and present-day nurseries and the coupling with (paleo)oceanographic conditions, are needed in order to fully evaluate the future global climatic and oceanographic alterations on the population stability of the GWS.

## Conclusions

The GWS, a top predator in today’s oceans, likely used the Coquimbo locality in Chile as a nursery, Pisco and Caldera as a feeding ground during the Pliocene. During this time, the GWS was more abundant in the south-eastern Pacific than it is today (Supplementary Table 1). Our results, added to the palaeontological and palaeoenvironmental evidence of the region, suggest that there were stable populations of GWS along the South American Pacific coast in the Cenozoic that recruited from at least one nursery area, raising new questions about the unusual presence for the modern GWS in the South Eastern Pacific population in contrast with the past.

## Methods

### Study area

The GWS has a fossil record that ranges from the lower Pliocene to the Pleistocene^37,38^. Our study is based on specimens from eight localities, one from southern Peru (Sacaco) and seven from Chile (Quebrada Camarones, La Cantera Baja, Quebrada Las Rosas, La Herradura, Mina Fosforita, Norte Bahia Caldera and Caldera: Fig. 1; Supplementary Dataset 1). Our sample set from Peru comes from the Sacaco locality (Pisco region, 15°S), which is part of the Pisco Formation. This formation ranges from the middle Miocene to the Pliocene^39^ and thus includes some of the oldest records of GWS. In Chile, the localities Caldera, Mina Fosforita and Norte Bahia Caldera (Caldera region, 27°S) belong to the Bahia Inglesa Formation. Recently, each major stratigraphic unit within this formation was dated using isotopes (^87^Sr/^86^Sr), giving a range from the middle Miocene to the lower Pleistocene^49^. The localities Quebrada Camarones, La Cantera Baja, Quebrada Las Rosas, La Herradura and La Cantera Baja (Coquimbo region, 29°S) are part of the Coquimbo Formation. Staig *et al*.^42^ proposed an age close to the middle Miocene-Late Pliocene for this geological formation. Due to the small sample sizes of fossil GWS teeth from certain localities (e.g., Norte Bahia Caldera), we treated the localities from the same region to a single locality (i.e., Pisco, Caldera and Coquimbo).

### Fossil data

We collected a total of 234 GWS fossil teeth: 69 from Coquimbo, 85 from Caldera and 80 from Pisco. Despite some uncertainties in the age of these lithostratigraphic units, all the specimens examined in this study are interpreted to have come from the Pliocene, because definitive *Carchardon carcharias* has not been recorded from pre-Zanclean rocks^37^ while exposures of post-Zanclean portions of those formations are limited^45^*. Carcharodon hubbelli*, that is interpreted to be a chronospecies with *C. carcharias*, is known from the late Miocene of Peru^38^, but all the teeth examined in this study exhibit well-developed serrations along the cutting edges of their crown characteristic of *C. carcharias*, (vs. *C. hubbelli* with weak serrations). Unlike most other Pliocene elasmobranch taxa with much smaller teeth that are easily overlooked during field surveys^37^, comparably large teeth of *C. carcharias* are generally readily noticeable in the field; thus, our collected sample sets of *C. carcharias* specimens that are represented by isolated teeth are assumed to represent random samples from different individuals of GWS.

### Tooth measurements and fossil body size estimates

For each tooth in lingual view, we measured the crown height (CH), crown width (CW), root height (RH), root width (RW), and total tooth height (TH) (Supplementary Dataset 1; Supplementary Fig. 1). We then estimated the total length (TL) of each individual based on the CH. Teeth are often used to extrapolate the body size of the GWS^62,63^, where the use of the tooth crown height is known to provide particularly reliable estimates about the total length of the species. The application of this approach to fossil specimens rely on the assumption that tooth allometry has remained stable in time. To do so, we used the allometric relationship between CH and TL in extant GWS^62^ where every tooth position in the jaw corresponds to one regression equation that expresses its TL. We consequently determined the position of each fossil tooth based on the overall morphology of the crown and root using illustrations from primary literature^64^ and following the tooth type nomenclature of Shimada^65^ (Fig. 2 and Supplementary Dataset 1). We assumed sexual differences in tooth allometry, if any at all, to be negligible because decisive sexual dimorphism in tooth morphology is not known for GWS^66^.

### Body size categories and analysis

Based on the size and life stages proposed by previous studies^43,44,48^, we classified the GWS into seven categories: (A) YOY ranging from 120 to 175 cm TL; (B) JWS ranging from 175 cm to 300 cm TL; (C) subadult measuring ≥300 to 360 cm for males and (D) ≥300 to 450 for females; and (E) adult measuring ≥360 cm TL for males and (F) ≥480 cm TL for females. Despite the size differences in the onset of sexual maturity between males and females, 450 cm TL is regarded as an average adult length^43,48^. Using the TL estimates derived from fossil teeth, we calculated the proportion of GWS specimens using the length categories defined by Ferguson^43^ and Bruce^44^ as follows: (A) YOY, (B) JWS, (C) subadult males, (D) subadult females, (E) adult females and (F) adult males and (G) “undetermined sex”. This approach assumes that life-history of the GWS has remained stable in time (i.e., the size of first maturity of fossil and modern populations is the same). We also evaluated whether TL distributions were composed of single or multiple modes (i.e. suggesting multiple size cohorts). The number of modes of the frequency distribution of TL was statistically calculated using a Gaussian finite mixture modelling (GMM)^67^, which evaluates whether a mixture of several distributions combine to create an observed frequency distribution. A Bayesian approach was used to estimate the optimum number of clusters or distributions, from one to nine, where larger Bayesian Information Criterion (BIC) values indicate stronger support for the model and number of clusters. Analyses were carried out using the library “mclust” in R^68^.

We defined the presence of palaeo-nurseries based on three main criteria: (a) the majority of specimens (>50%) are juveniles, (b) locality interpreted as shallow marine environment and (c) food availability (i.e. potential preys)^16,17^.

## Supplementary information

Supplementary Information.

Supplementary Information2.

## Data Availability

All relevant data are within the paper and its Supporting Information files.
