# Supplementary material for: First evidence of a palaeo-nursery area of the great white shark
Source: Sci Rep. 2020 May 22;10:8502. doi: 10.1038/s41598-020-65101-1 (PMC7244757; doi:10.1038/s41598-020-65101-1)
Supplement: Supplementary file 2 — Supplementary Information2. [file 41598_2020_65101_MOESM2_ESM.docx]

**First evidence of a palaeo-nursery area of the great white shark**

Jaime A. Villafaña^1,2*^, Sebastian Hernandez^3,4^, Alonso Alvarado^3^, Kenshu Shimada^5,6^, Catalina Pimiento^7,8^, Marcelo M. Rivadeneira^9,10,11^, and Jürgen Kriwet^1^

Affiliations

^1^University of Vienna, Department of Palaeontology, Althanstraße 14, Geocenter, 1090 Vienna, Austria, ^2^Centro de Investigación en Recursos Naturales y Sustentabilidad, Universidad Bernardo O'Higgins, Santiago, Chile, ^3^Biomolecular Laboratory, Center for International Programs, Universidad VERITAS, 10105 San José, Costa Rica, ^4^Sala de Colecciones Biológica, Facultad de Ciencias del Mar, Universidad Católica del Norte, Coquimbo, Chile, ^5^Department of Environmental Science and Studies and Department of Biological Sciences, DePaul University, Chicago, Illinois 60614, USA, ^6^Sternberg Museum of Natural History, Hays, Kansas 67601, USA, ^7^Department of Biosciences, Swansea University, Swansea SA28PP, United Kingdom, ^8^Smithsonian Tropical Research Institute, Balboa, Panama. ^9^Laboratorio de Paleobiología, Centro de Estudios Avanzados en Zonas Áridas (CEAZA), Coquimbo, Chile, ^10^Departamento de Biología Marina, Facultad de Ciencias Biológicas, Universidad Católica del Norte, Larrondo 1281, Coquimbo, Chile, ^11^ Departamento de Biología, Universidad de La Serena, Av. Raul Bitrán 1305, La Serena, Chile.

*corresponding author: villafanaj88@univie.ac.at


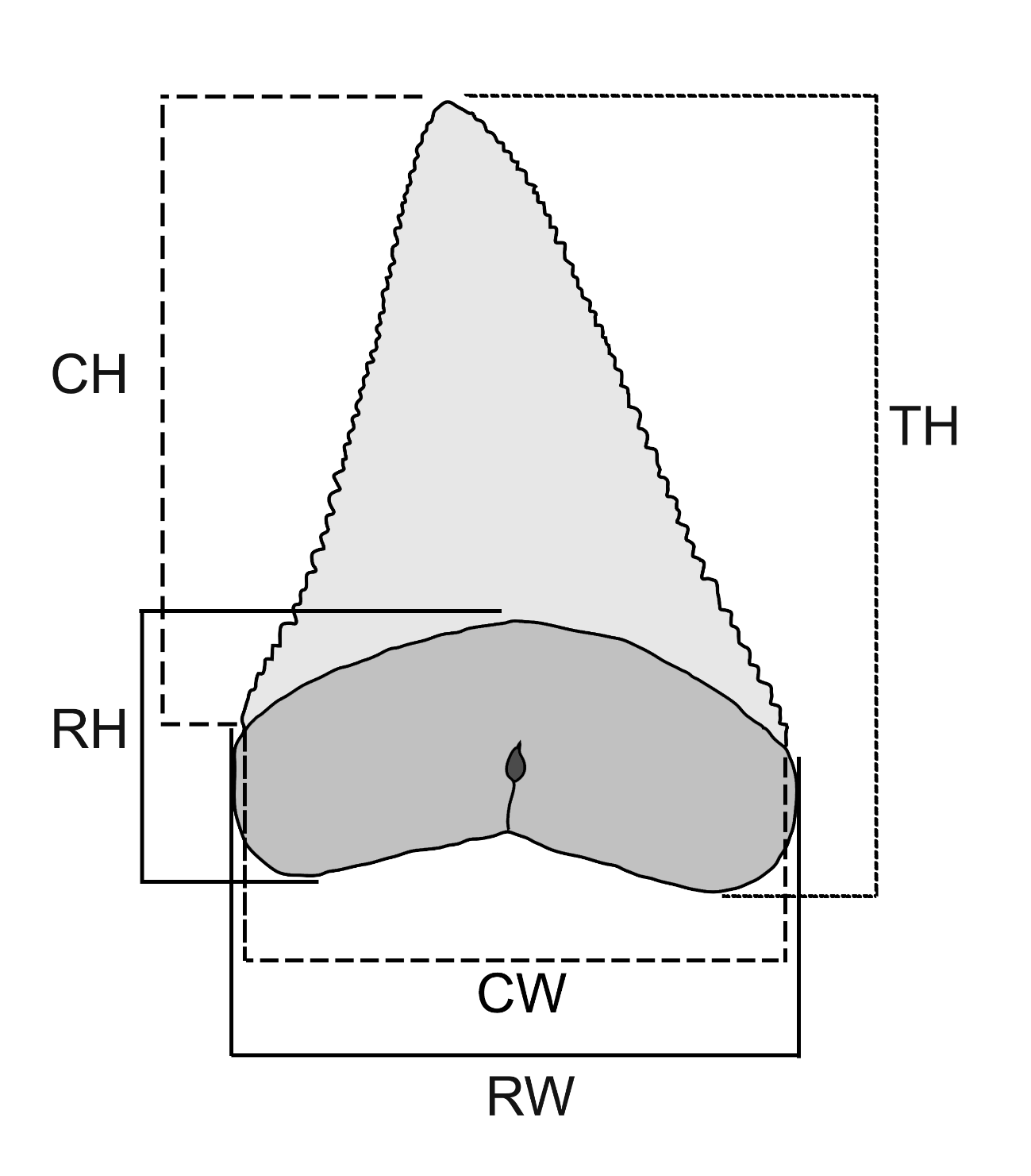


**Supplementary Figure 1.** Tooth measurement codes. CH: crown height, CW: crown width, RH: root height, RW: root width, TL: total length.


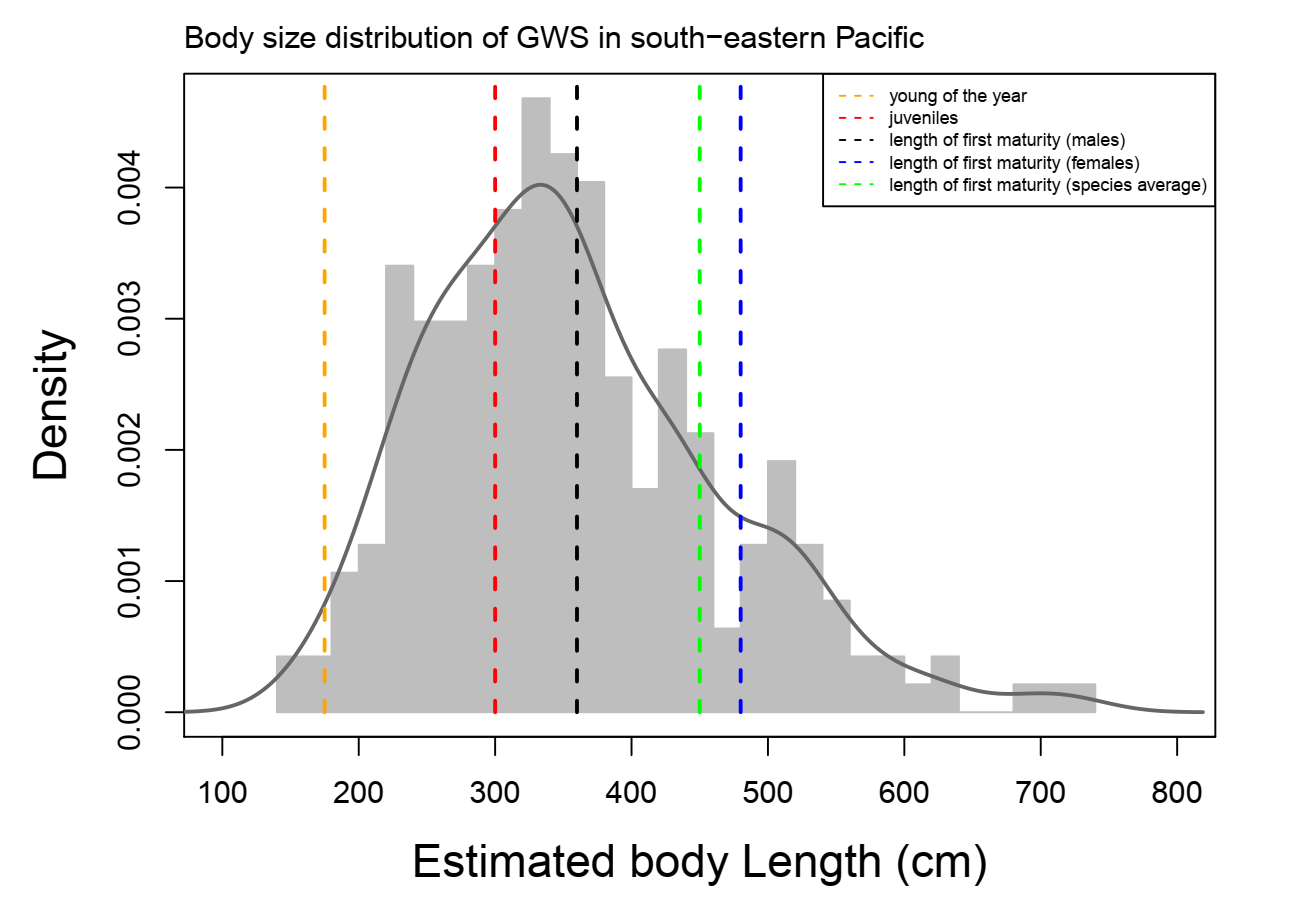


**Supplementary Figure 2**. Frequency distribution of the estimated body length of GWS from the eastern Pacific of South America. Dashed lines represent the length of young of the year, juveniles, first maturity for males, females and species average.


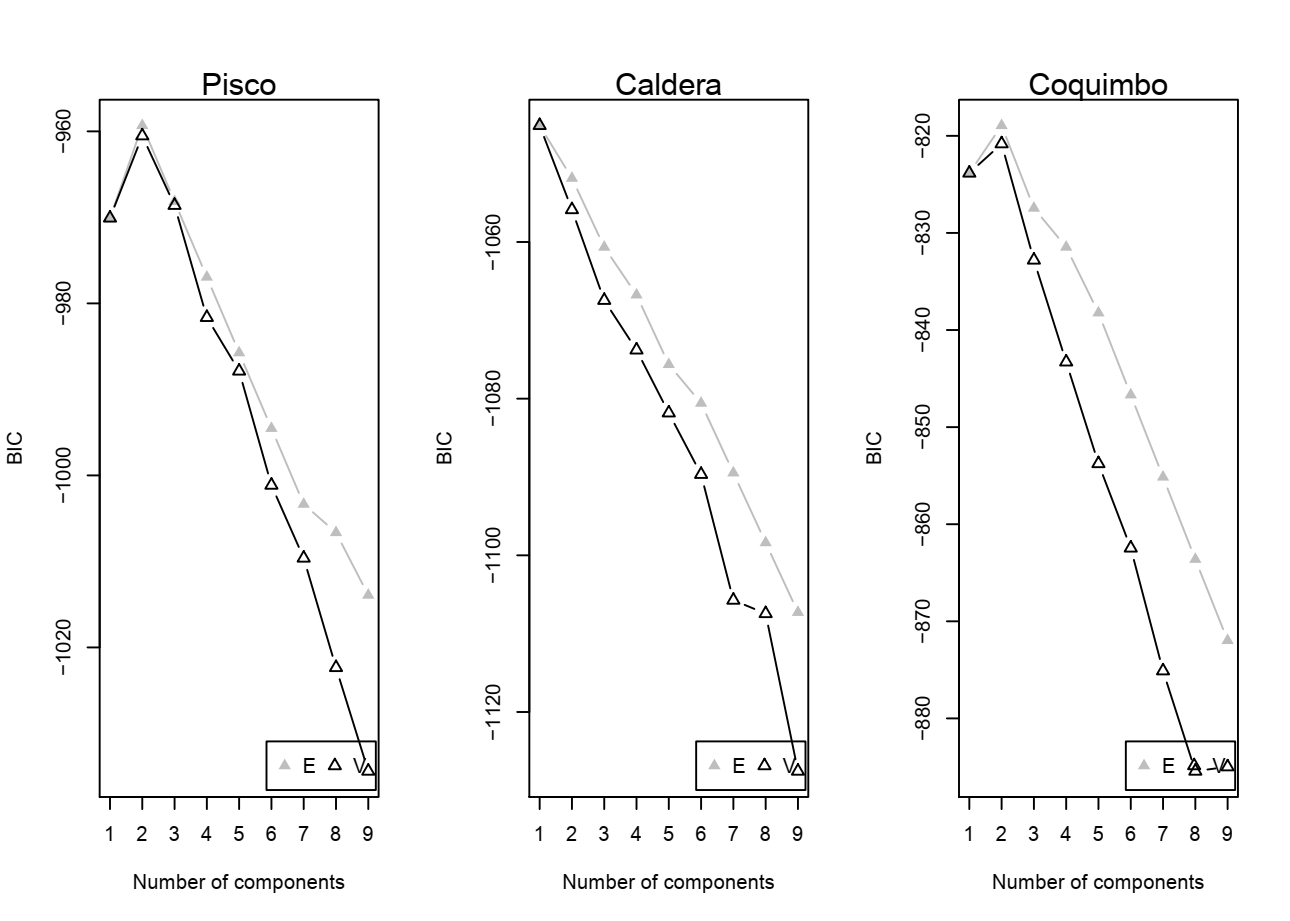


**Supplementary Figure 3.** BIC profiles of finite Gaussian Mixture Models to detect the presence of multiple modes in the body size frequency distribution of the great White shark in the three studied regions. Analyses based on two algorithms (E: equal variance; V; variable or unequal variance). Based on BIC profiles, a two-component model is suggested for Pisco and Coquimbo, a single cluster for Caldera.

| **Date** | **Location** | **Specific geographic location** | **Fatal (F) / Non-Fatal (NF) / Landed specimen (LE)** | **Reference** |
| --- | --- | --- | --- | --- |
| 1949 (specific date not provided) | Miraflores, Lima, Perú | Nearby Miraflores | LE | Schweigger^31^ |
| August 28, 1944 | Ancon Bay, Lima, Perú | off Ancon Bay | LE | Hildebrand^29^ |
| September 29, 1963 | El Panul, Coquimbo, Chile | 30° 01’ S, 71° 23’ W | F | Cea & McCosker^32^ |
| January 5, 1980 | Punta Negra, Los Molles, Chile | 32° 08’ S, 71° 32’ W | F | Balbontin & Reyes^33^, Cea y McCosker^32^ |
| December 15, 1984 | Punta Lobos, Los Vilos, Chile | 31° 56’ S, 71° 31’ W | F | Hernandez & Lamilla^30^ |
| March 23, 1994 | Salas and Gómez Island | 36 miles E Salas and Gomez Island | NF | Hernandez & Lamilla^30^ |
| May 27, 1987 | Río Loa, Iquique, Chile | 12 km offshore Loa River mouth | RC | Hernandez & Lamilla^30^ |
| 1970s (specific date not provided) | Coquimbo Bay, Coquimbo, Chile | Nearby Caleta Peñuelas | RC | Hernandez & Lamilla^30^ |

**Supplementary Table 1.** Recent GWS reports from the eastern Pacific
